# Next-Generation Sequencing Identifies Transportin 3 as the Causative Gene for LGMD1F

**DOI:** 10.1371/journal.pone.0063536

**Published:** 2013-05-07

**Authors:** Annalaura Torella, Marina Fanin, Margherita Mutarelli, Enrico Peterle, Francesca Del Vecchio Blanco, Rossella Rispoli, Marco Savarese, Arcomaria Garofalo, Giulio Piluso, Lucia Morandi, Giulia Ricci, Gabriele Siciliano, Corrado Angelini, Vincenzo Nigro

**Affiliations:** 1 TIGEM (Telethon Institute of Genetics and Medicine), Napoli, Italy; 2 Dipartimento di Biochimica Biofisica e Patologia Generale, Seconda Università degli Studi di Napoli, Napoli, Italy; 3 Dipartimento di Neuroscienze, Università degli Studi di Padova, Padova, Italy; 4 Cancer Research UK, London, United Kingdom; 5 Fondazione IRCCS Istituto Neurologico C. Besta, Milano, Italy; 6 Dipartimento di Medicina clinica e sperimentale, Università degli Studi di Pisa, Pisa, Italy; 7 IRCSS S. Camillo, Venezia, Italy; Medical College of Georgia, United States of America

## Abstract

Limb-girdle muscular dystrophies (LGMD) are genetically and clinically heterogeneous conditions. We investigated a large family with autosomal dominant transmission pattern, previously classified as LGMD1F and mapped to chromosome 7q32. Affected members are characterized by muscle weakness affecting earlier the pelvic girdle and the ileopsoas muscles. We sequenced the whole exome of four family members and identified a shared heterozygous frame-shift variant in the Transportin 3 (*TNPO3*) gene, encoding a member of the importin-β super-family. The *TNPO3* gene is mapped within the LGMD1F critical interval and its 923-amino acid human gene product is also expressed in skeletal muscle. In addition, we identified an isolated case of LGMD with a new missense mutation in the same gene. We localized the mutant TNPO3 around the nucleus, but not inside. The involvement of gene related to the nuclear transport suggests a novel disease mechanism leading to muscular dystrophy.

## Introduction

Limb girdle muscular dystrophies (LGMDs) are characterized by a progressive weakness that begins from the proximal limb muscles, due to a number of independent genetic defects that are distinct from the X-linked Duchenne and Becker muscular dystrophies [1], [2]. In addition to the genetic heterogeneity, the different forms are clinically heterogeneous, with the age at onset of symptoms varying from early childhood to late adulthood [3]. The milder the symptoms are, more difficult is the LGMD diagnosis. Magnetic resonance imaging is helpful to characterise the severity and pattern of muscle involvement [4], [5], but recognition of LGMD type might be hard [6].

Muscle biopsy of LGMD patients generally shows a diffuse variation in fiber size, necrosis, regeneration and fibrosis, but the degree of these factors is variable and does not parallel the clinical severity. Based on the histological features alone, there is scarce, if any, possibility of diagnosing a specific LGMD form, but western blot and immunofluorescence can address to the true defect that can be demonstrated by the finding of a mutation in the corresponding gene.

The primary distinction is made between the autosomal dominant (LGMD1) and the autosomal recessive forms (LGMD2), with an alphabet letter indicating the order of gene mapping [2]. Eight LGMD1 loci have been identified so far. At present, only four autosomal dominant LGMD genes are known, encoding Myotilin (LGMD1A), Lamin A/C (LGMD1B), Caveolin-3 (LGMD1C), and DNAJB6 [7], [8] (LGMD1D). Some patients with mutations in these four genes fulfill the diagnostic criteria for the LGMDs, but others show a much wider spectrum of different phenotypes. LGMD1F is a very puzzling disease [9]. It is characterized by muscle weakness affecting earlier the pelvic girdle and especially the ileopsoas muscle. Interestingly, some patients presented with a juvenile-onset form. In the original article [9], rimmed vacuoles were reported. Recently, immunofluorescence and ultrastructural studies pointed to the presence of large protein aggregates and autophagosomes [10]. Many alterations of myofibrillar component were also detected [10]. The critical interval was mapped to a 3.68-Mb interval on chromosome 7q32.1–7q32.2 [11]. Given the size of the kindred and the very accurate linkage analysis, the gene identification has been considered within reach. In this region the obvious candidate is the *FLNC* (Filamin C) gene that is mutated in a form of autosomal dominant myofibrillar myopathy (MFM) with limb-girdle involvement [12], as well as in a second form characterized by the weakness of distal muscles and non-specific myopathic features [13]. However, the early onset of some LGMD1F and the lack of massive protein aggregates of MFM suggest that LGMD1F may be a different disorder: despite a thorough search, no mutation was found in the *FLNC* gene [11]. In addition, other candidate genes of the region were excluded and LGMD1F remained unsolved for many years.

In the last few years, the techniques of next-generation sequencing (NGS) coupled with target enrichment protocols enhanced the molecular genetic diagnostics [14], [15]. We studied the original Spanish family with additional family members by exome sequencing [16] using two different NGS platforms. We sequenced the whole exome of four affected individuals and identified a number of new variations, one of which was completely new, shared by all affected subjects, and mapped to 7q32.

## Methods

### Ethics Statement

This study adhered to the tenets of the Declaration of Helsinki. Subjects for this study were recruited at Padua University and exome analysis was performed at the Second University of Naples and at the Telethon Institute of Genetics and Medicine. Participants were informed of the nature and risks of the study, and signed consent forms were obtained. The institutional review board of the Second University of Napoli (SUN) reviewed and approved this study (prot. AOP-SUN 862).

### Patients

Nineteen patients were included in the clinical study, and they all fulfilled the diagnostic criteria for LGMD that include a characteristic pattern of muscular weakness primarily affecting pelvic girdle, assessed according to MRC Scale and a modified Gardner-Medwin & Walton scale for proximal LGMD. Age at onset was assessed as described. We collected blood from 19 patients and 8 healthy relatives. Skeletal muscle biopsy from the deltoid or *vastus lateralis* was taken from 2 affected individuals.

### Exome sequencing and analysis

Enrichment was performed by hybridization of shotgun fragment (average size 141 bp) libraries to Agilent SureSelect Human All Exon 50 Mb (Agilent Technologies, Santa Clara, CA, USA) in-solution capture assays. Using the SOLiD system v4 (Life Technologies), we generated an average of 4.2 Gb of mappable sequence data per sample to achieve ∼20x mean coverage of the targeted exome. The sequences were analyzed using an automated custom pipeline designed to perform every step of the analysis with the appropriate program or custom script. Sequencing reads were first colour-corrected using SOLiD Accuracy Enhancer Tool (SAET), then mapped to the reference genome (UCSC, hg19 build) using the software BioScope v1.3 (Life Technologies, Carlsbad, CA, USA) and duplicate reads were removed using Picard (http://picard.sourceforge.net). Single nucleotide variations (SNV) and in-del mutation calling analyses were carried out using the diBayes algorithm with medium stringency settings and the SOLiD Small Indel Fragment Tool (www3.appliedbiosystems.com), respectively.

One of the samples was sent to a commercial provider (Otogenetics Corporation, Norcross, GA, USA) who performed both whole exome enrichment with the SeqCap EZ Human Exome Library v2.0 (Roche NimbleGen, Inc, Madison, WI, USA) and sequencing with the HiSeq2000 platform (Illumina inc., San Diego, CA, USA). The sequences were analyzed using another automated pipeline designed to handle Illumina data with custom scripts and publicly available software. Paired sequencing reads were aligned to the reference genome (UCSC, hg19 build) using BWA [17] and post-alignment process and duplicate removal was performed using SAMtools [18] and Picard. Further processing (local realignment around in-del and base recalibration) and SNV and in-del calling were performed with Genome Analysis Toolkit (GATK) [19].

The called SNV and in-del variants produced with both platforms were annotated using ANNOVAR [20], the relative position in genes using RefSeq [21], amino acid change, presence in dbSNP v137 [22], frequency in NHLBI Exome Variant Server (http://evs.gs.washington.edu/EVS) and 1000 genomes large scale projects (http://www.1000genomes.org) [23], conservation and different prediction algorithms of damaging effect on protein activity [24], [25], [26], [27] and conservation scores [28], [29]. The annotated results were then imported into an in-house variation database, also used to make comparisons among samples and filter results. The alignments at candidate positions were visually inspected using the Integrative Genomics Viewer (IGV)[30]. The accession number of the dataset of this study is ERP002413 (Sequence Read Archive – EBI at www.ebi.ac.uk).

### Mutation Detection

We designed both cDNA and intronic primers to amplify the cDNA and the 22 coding exons plus the 3’UTR exon of the TNPO3 gene (MIM 610032; NM_012470.3, NM_001191028.2) (Table S2). In addition, we sequenced all the other exons at the disease interval that were inadequately covered (<10x) (Table S3). We also designed additional primers to map the alternatively spliced products. We purified the amplicons and sequenced them by using the fluorescent dideoxyterminator method on an automatic sequencer (ABI 3130XL).

### Immunoblotting Analysis

For TNPO3 immunoblotting, muscle samples were homogenized in a lyses assay buffer (Urea 8 M, SDS 4%, 125 mM Tris HCl pH 6.8). The samples were separated on sodium dodecyl sulphate –9% polyacrylamide gel electrophoresis and transferred to nitrocellulose membrane. After blocking in 10% no fat dry milk in Tween-Tris-Buffered Saline (TTBS-1X) buffer (10 mM Tris-HCl, 150 mM NaCl, 0.05% TWEEN 20) for 1h, the membranes were incubated with primary antibodies in TTBS 1X at room temperature for 2 h. The monoclonal antibody, recognizing a recombinant fragment (Human) from near the N terminus of TNPO3, was used in this experiment with a 1∶100 dilution (Abcam®). We also used the rabbit monoclonal antibody Anti-TNPO3 antibody [EPR5264] (ab109386) that recognizes a synthetic peptide corresponding to residues near the C terminal of Human TNPO3. This was used for WB at 1∶300 dilution.

Following primary antibody incubation and rinses, the membranes were incubated with the secondary antibody, goat anti-mouse immunoglobulin conjugated with horseradish (Sigma), with 1∶10,000 dilution in 0.5% dry milk and TTBS 1X. After 45 minutes of antibody incubation and five washes with TTBS 1X buffer, the TNPO3 protein band was visualized with a chemiluminescence reagent (Supersignal, WestPico, Pierce) and exposed to X-ray film.

To perform this analysis, Coomassie blue staining was used for the evaluation of the myosin protein expression to understand the variations in the levels of the proteins loaded.

### Transfection

Plasmid pcDNA6/A encoding N-terminal HA-tagged *TNPO3* full length was obtained by NR Landau, NewYork University School of Medicine [31]. We subcloned (*EcoRI-NheI*) HA-TNPO3 exons 1 to 17 in pCS2+ and exons 17 to 22 or 23 were amplified by PCR from cDNA and cloned in pCS2+/HA-TNPO3_1-17 (NheI-XhoI). Four human *TNPO3* cDNA constructs were cloned into the pCS2HA plasmid : 1) Wt TNPO3 isoform with 22 exons; 2) TNPO3 isoform with 22 exons containing del A p.X924C; 3) Wt TNPO3 isoform with 23 exons; 4) TNPO3 isoform with 23 exons containing del A p.X924C. We used 500 ng for transient trasfection of HeLa cells (2×10^5^) cells using PolyFect Transfection Reagent (Qiagen) according to manifacturer’s instruction. Cells were grown on glass coverslip put into 12 well plates. They were cultured in Dulbecco’s modified eagle’s medium (DMEM) supplemented with 10% (v/v) foetal bovine serum and penicillin-streptomycin (GIBCO-Invitrogen) and maintained in a 5% CO2 incubator at 37°C. 48 hours after transfection, cells were fixed with 4% paraformaldehyde in PBS for 10 min at RT, permeabilized in 0,2% Triton X-100 in PBS for 5 min at RT, and blocked for 1 h in Blocking solution (BSA 6%, Horse Serum 5% in PBS). Cells were incubated for 1 h at RT with primary antibodies, followed by 1 h incubation at RT with FITC-conjugated anti-rabbit and/or Cy3-conjugated anti-mouse antibodies.

## Results

### Exome analysis

**Figure 1:**
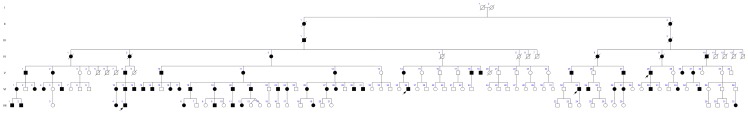
LGMD1F family pedigree. Squares represent male; circles represent female; white figures symbolize normal individuals; black figures indicate individuals with clinical muscular dystrophy. The original LGMD1F family has been extended from subject II,2 and now includes 64 LGMD patients of both sexes and five non-penetrant carriers (IV-4, V-26, V-29, V-33, and VI-68). The whole-exome sequencing was performed in four patients indicated by arrows (V-28, VI-36, VI-53, VII-5).

The original LGMD1F family has been extended (Figure 1) to include additional family members in seven generations starting from subject II, 2. The updated pedigree includes 64 LGMD patients of both sexes and five non-penetrant carriers (93% penetrance). To perform an informative exome sequencing analysis, we selected four affected family members (VII-5, VI-53, V-28, and VI-36) with a manifest LGMD phenotype separated by the largest number of meioses. Interestingly, two family members (VI-53 and V-28) were absent from the original family used for the linkage analyses. DNA samples of three individuals (V-28, VI-53, VII-5) were fragmented, enriched using the SureSelect whole exome kit and sequenced by SOliD. DNA, muscle RNA and proteins were extracted for the studies. We found ∼20,000 exonic variations for each sample, 5,722 of which were common to all three (Table 1 and Table S1) of which 2,471 were non synonymous. Considering the dominant mode of inheritance of LGMD1F, we focused on the heterozygous calls and discarded all variants present with a frequency higher than 1% in the NHLBI Exome Variant Server (http://evs.gs.washington.edu/EVS) or 1000genomes [32] large scale projects. The resulting filtered list of 273 variants was composed of 253 missense, 14 stopgain, 2 frameshift deletions, 2 nonframeshift insertions/deletions and 2 stoploss variations. Only two variants were mapped into the disease interval between D7S1822 and D7S2519 (positions: 126,287,140-129,964,025) [11]: a nonsynonymous SNV in the gene IRF5 and a frame-shift deletion that modify the termination codon in the exon 22 (stoploss) in the TNPO3 on chromosome 7q32.1 at position 128,597,310 (GRCh37/hg19). To verify whether we could have missed by NGS other shared variants, we resequenced by the dideoxy-chain termination method all the coding exons and flanking introns of the full 7q32 region with lower/absent coverage (Table S3). No other shared unknown variant was found. In addition, the DNA sample of VI-36 was sent to a commercial provider for exome sequencing using the Illumina platform HiSeq2000. Among 153 variations that were shared by all, the only one in the disease interval was that in the TNPO3 gene (Table 1). Interestingly, this was the only variation of the whole exome that resulted absent in dbSNP137. We also refined the interval: the SNP rs45445295 at the SMO gene at position 128,845,555 was present in some affected members (V-8, VI-60, V-14, VI-11, V-25, V-12), but it was absent in other affected members (VI-57, VI-27, VI-56) and in all non-affected individuals. Therefore, the linked region associated with disease locus was ∼1.1 Mb smaller (126,287,140-128,845,555) than that reported by Palenzuela [11].

**Table 1:** Total and Shared Variants in Patients with LGMD1F.

Patient variant type	V-28	VI-53	VII-5	Shared by all SOLiD	VI-36	Shared by all four
exonic/splicing	21,105	21,366	17,123	5,722	17,183	**4,212**
non synonymous	11,852	11,713	9,051	2,471	7,831	**1,687**
heterozygous	9,348	9,138	6,812	644	4,693	**153**
frequency in EVS and 1000genomes<1%	6,102	5,785	3,860	273	486	**10**
Within LGMD1F interval	13	11	5	2	1	**1**

**Figure 2:**
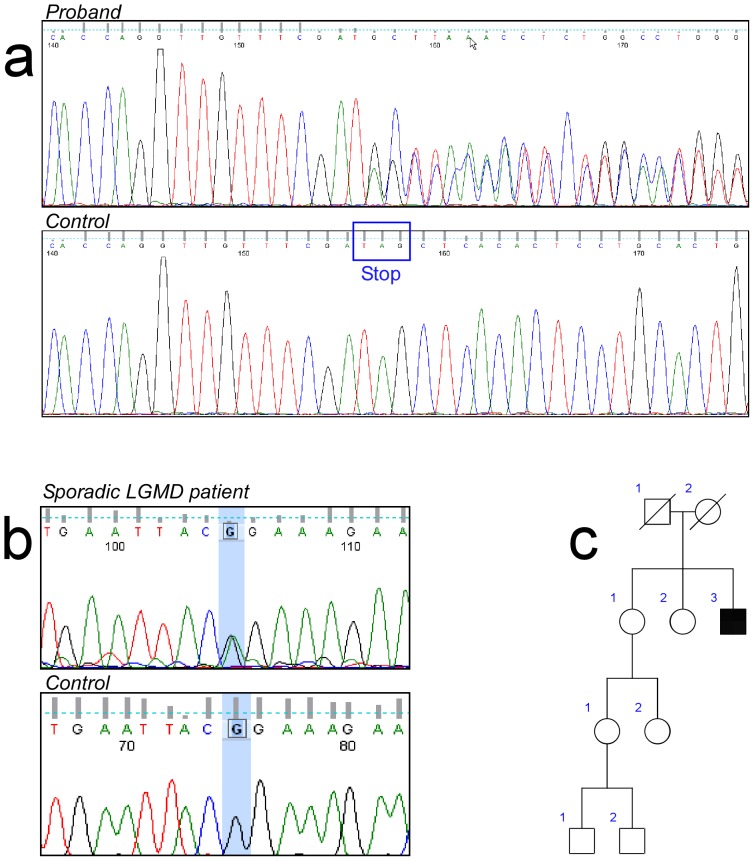
Sequence analyses of the TNPO3 mutations. a) Heterozygous delA mutation in Exon 22 of the *TNPO3* gene in Proband VII-5. Aligned electropherograms show mutated (top) and wild-type (bottom) sequences; b) Heterozygous. c.G2453A) in exon 21 of the *TNPO3* gene; c) Pedigree of the isolated case.

To confirm the complete co-segregation of the nonstop *TNPO3* variant with LGMD1F, we analyzed all available family members, affected and non-affected. We sequenced by the Sanger method all the samples and, in addition, we took advantage of an *AluI* restriction site that was lost upon mutation. We observed the complete co-segregation of the *TNPO3* variant with the disease (Figure 2a and Table S4).

We extended the analysis to additional 64 samples from LGMD1 and isolated LGMD cases, using a next generation sequencing approach. In particular, we performed a custom enrichment of exons of genes involved in muscular dystrophies, including *TNPO3*.

In a single individual, we found a heterozygous G>A transition (c.G2453A) in exon 21 of the *TNPO3* gene. This point mutation changes the Arginine in position 818 with a Proline (Figure 2b). This is an extreme conserved residue that is predicted to be damaging by all the used bioinformatic tools (SIFT, PolyPhen, Mutation Taster and LRT). Moreover, the variation is not listed in dbSNP and in the other recently developed databases collecting NGS data (Exome Variant Server and 1,000 genomes database) neither in our internal database of 150 samples whose exomes have been sequenced in our lab.

This variation has not been found in the healthy sister (Figure 2c). In addition, this patient bears no other major mutation in other 98 “muscular-disease” genes, but a single heterozygous ANO5 variation (Glu95Lys), without a clear significance. Young adult onset has been observed in this patient, showing a characteristic LGMD phenotype. Muscular histopathological data evidenced dystrophic features and, in addition, discrete mitochondrial alterations, with sporadic ragged-red fibers and cytochrome c oxidase negative fibers. Mutations in the mitochondrial DNA were excluded.

### Importance of the TNPO3 mutation

To analyze the effects of the nonstop mutation on *TNPO3* gene products, we first performed the mRNA analysis using skeletal muscle biopsy of a patient compared with a normal control. In both cases, we identified two differently spliced muscular versions of the gene, both including exon 22. Form A that also join exon 22 to exon 23 that is non coding and form B that ends in exon 22. These forms encode the same protein, when the DNA sequence is normal, because the stop codon is in exon 22. However, the LGMD1F mutation eliminates this stop and, for both forms, the muscle protein product is extended by the frame-shift. Form A should be 15 amino acids longer (CSHSCSVPVTQECLF), while form B should contain additional 95 amino acids.

**Figure 3:**
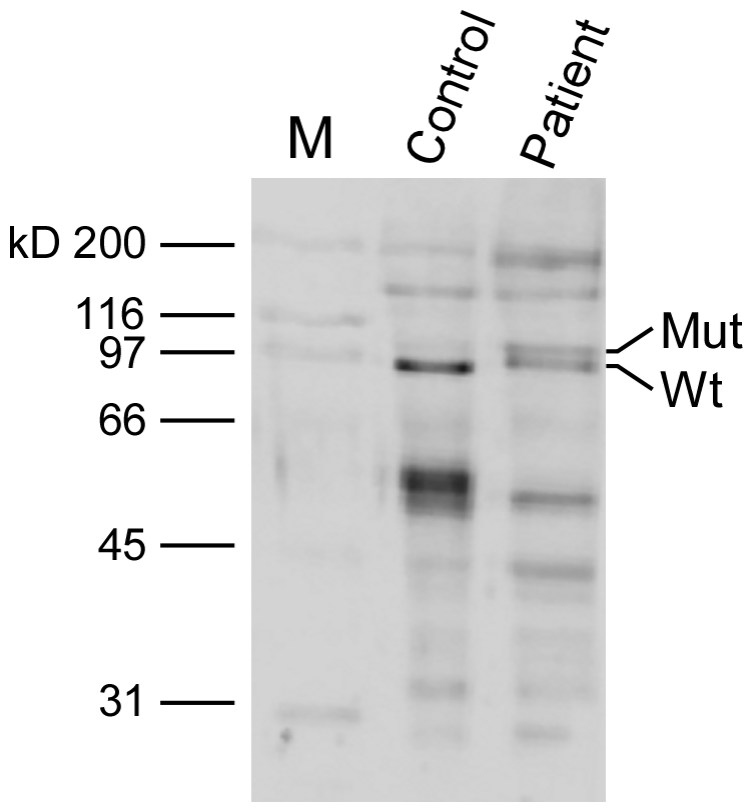
Western blot analysis of skeletal muscle tissue with antibodies to TNPO3. Equal amounts of muscle proteins from a LGMD1F patient and a control were run in each lane (10 µg) on a 9% SDS-polyacrylamide gel and then blotted onto nitrocellulose membrane. In this experiment, we used a monoclonal antibody that recognizes a recombinant fragment (Human) near the N terminus of TNPO3 at a 1∶100 dilution. A double band is visible in the patient only.

We then performed immunoblotting analyses of the skeletal muscle biopsy using the anti TNPO3 antibody. While mutant form A is virtually overlapping with wild-type form A, a mutant form B can be appreciated by western blot analysis of muscle samples as a higher molecular weight band (Figure 3).

**Figure 4:**
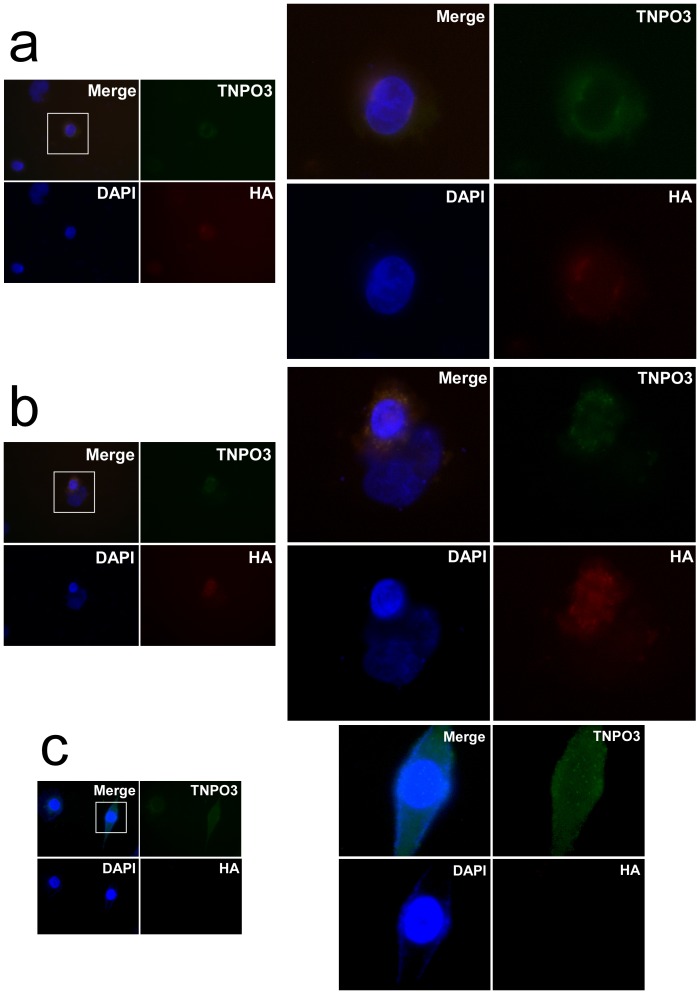
Indirect immunofluorescence analysis of the wt-hTNPO3 compared with delA p.X924C -hTNPO3. Following transient transfections, HeLa cells were incubated for 48 h with normal DMEM and detected by anti-HA immunofluorescence. Nuclei are stained with DAPI (blue). The endogenous protein is recognized using a rabbit monoclonal anti-TNPO3 antibody (green), while the transfected TNPO3 proteins were HA-tagged (red). **a**) An accumulation around the nucleus is usually observed using the mutant delA p.X924C -hTNPO3. **b**) The typical intranuclear staining pattern can be observed in cells transfected with wt-hTNPO3 (in red) or **c)** in non transfected HeLa cells.

We generated a construct expressing the WT and del A p.X924C allele. HeLa cells were transfected with either the Wt or the mutant TNPO3. The transfected proteins were distinguished from the endogenous TNPO3 by adding a HA-tag. Figure 4 shows that the WT TNPO3 entered the nucleus, while the mutant was usually around the periphery of the nucleus.

## Discussion

Here, we report the identification of a frame-shift variant del A p.X924C at the *TNPO3*/Transportin-SR2 gene on chromosome 7q32.1 at position 128,597,310 (GRCh37/hg19) in all patients with limb girdle muscular dystrophy 1F. No other variant was shared by four affected members of the family. The variant modifies the true stop codon and encode for two elongated proteins of 15 and 95 amino acids. Interestingly, one flanking SNP (rs12539741 at 128,596,805) has been identified in association with others in the region as a susceptibility locus for primary biliary cirrhosis [33]. Considering that the affected family members may share a ∼2.6 Mb-region on chromosome 7q32, there is the possibility that any other rare heterozygous variant could co-segregate in *cis* with the true LGMD1F mutation. Thus, we searched in a large collection of patients independent *TNPO3* disease-associated variations. We found a missense Arg818Pro in an isolated LGMD case co-segregating with the disease in this family. This variation was predicted as causative by the nature of the change and the conservation.

Transportin 3 is a member of the importin β super-family that imports numerous proteins to the nucleus, including serine/arginine-rich proteins (SR proteins) that control mRNA splicing [34], [35]. Transportin 1 (TNPO1), also known as karyopherin β-2, mediates the nuclear import of M9-bearing proteins[36], while TNPO2 (karyopherin β -2B) participates directly in the export of a large proportion of cellular mRNAs[37]. There are two main TNPO3 proteins: variant 1 that is 923 amino acids long and variant 2 composed of 859 amino acids, while a longer variant of the 3’ terminus (hTNRSR1[35]) was probably due to a sequence artifact. The 923-amino acid protein is found in the skeletal muscle, translated from two equivalent messengers that include or not the 3’ noncoding exon.

The *TNPO3* nonstop allele hindered the nuclear localization of the protein in HeLa cells. Given the role of TNPO3 protein in the nuclear export/import of proteins and in the RNA splicing mechanism, we have two hypotheses: 1) this mutation blocks the nuclear export/import because the longer protein is unable to move to the nucleus, but remains outside the nuclear membrane 2) the mutated protein does not interact with the cargo proteins, causing the block of the nuclear import/export.

Our present data indicate that *TNPO3* is the gene mutated in LGMD1F. Additional functional studies in model organisms are, however, necessary to understand whether the dominant role of these mutations is due to haploinsufficiency or to a dominant-negative mechanism. This should be possible by the use of antisense morpholino oligos in *Danio rerio* (Zebrafish) where a single and conserved *TNPO3* ortholog is present with 792/923 (86%) amino acid identity (Table S5).

Advances in the knowledge of limb-girdle muscular dystrophies have been made in the last few years. With LGMD1F, five different autosomal dominant LGMD genes have been so far recognized. The use of NGS technologies promises a revolution in diagnostics and a more rapid characterization of patients.

## Supporting Information

Table S1 Exome sequencing data.
(DOC)

Table S2 Primers designed for *TNPO3* amplification and Sanger sequencing.
(DOC)

Table S3 Exons inadequately covered by NGS exome sequencing and primers designed for Sanger sequencing.
(DOC)

Table S4 Co-segregation study.
(DOC)

Table S5 Alignment of Human and *Danio r.* TNPO3 proteins.
(DOC)

Table S6 Shared SNVs in the four samples sequenced by NGS.
(XLSX)
