# Supplementary material for: Next-Generation Sequencing Identifies Transportin 3 as the Causative Gene for LGMD1F
Source: PLoS One. 2013 May 7;8(5):e63536. doi: 10.1371/journal.pone.0063536 (PMC3646821; doi:10.1371/journal.pone.0063536)
Supplement: Table S1 — Exome sequencing data. (DOC) [file pone.0063536.s001.doc]

**Table S1**: Exome sequencing data

| ***Sample*** | ***V-28*** | ***VI-53*** | ***VII-5*** | ***VI-36*** |
| --- | --- | --- | --- | --- |
| *Platform* | SOLiD | SOLiD | SOLiD | Illumina |
| *Enrichment* | SureSelect All Exon 50Mb | SureSelect All Exon 50Mb | SureSelect All Exon 50Mb | SeqCap EZ Exome v2 |
| *Library* | Single read | Single read | Single read | Paired end |
| *Gb produced* | 4.0 | 3.8 | 4.7 | 4.3 |
| *Total Reads* | 79,626,531 | 75,581,330 | 94,187,212 | 47,236,251 |
| *Mapped Reads* | 49,215,512 | 49,203,293 | 53,422,231 | 41,853,243 |
| *Duplicated Reads* | 20,663,731 | 22,024,635 | 21,839,257 | 3,167,881 |
| *Informative Reads* | 28,557,304 | 27,183,902 | 31,588,924 | 38,685,362 |
| *Not Covered* | 10,77% | 10,31% | 11,36% | 4,82% |
| *Covered ≥ 1X* | 89,23% | 89,69% | 88,64% | 95,18% |
| *Covered ≥ 10X* | 64,61% | 66,25% | 65,29% | 71,94% |
| *Covered ≥ 20X* | 39,48% | 42,57% | 40,54% | 45,48% |
| *Read length (bp)* | 50 | 50 | 50 | 90 |
