# Supplementary material for: Next-Generation Sequencing Identifies Transportin 3 as the Causative Gene for LGMD1F
Source: PLoS One. 2013 May 7;8(5):e63536. doi: 10.1371/journal.pone.0063536 (PMC3646821; doi:10.1371/journal.pone.0063536)
Supplement: Table S2 — Primers designed for TNPO3 amplification and Sanger sequencing. (DOC) [file pone.0063536.s002.doc]

**Table S2**: Primers designed for *TNPO3* amplification and Sanger sequencing

| **Genomic primers** | |
| --- | --- |
| TNPO3ex1F | GACAGTGAGGAAGCGCGAAG |
| TNPO3ex1R | CCAAAAGCGGCAAAGATGTAG |
| TNPO3ex2F | CATAGCCTGCCTCATATTCATCAC |
| TNPO3ex2R | CATTGTTCTTCCCCTCTGCTAAAC |
| TNPO3ex3F | TGTTGAACAGGGCCTATAACAATG |
| TNPO3ex3R | TGGCCTAGCAATACGAATTAAAAC |
| TNPO3ex4F | TAGAATTGGAGGTTGGGAGAAATG |
| TNPO3ex4R | TTTGCTGCAATTAATTCAAGAAGTG |
| TNPO3ex5F | CTTAACAGTGGTGGGAATGTTGC |
| TNPO3ex5R | CCAAAGCATCATCATTAAATTTTGC |
| TNPO3ex6F | TTGGCTGTTTTATTGCCATCTTTC |
| TNPO3ex6R | CTGCATTCTTAATACACGCCTTCC |
| TNPO3ex7F | CAGGGCCAACTGTAGATCACTC |
| TNPO3ex7R | CTGAGAATCACAAATGACAGACGA |
| TNPO3ex8F | CATGGGGATTGTGATACAGCATAG |
| TNPO3ex8R | TGGGCACGAGGTAATGAAATATAG |
| TNPO3ex9F | AGGTGACTTCTTAACTGGCTCCAC |
| TNPO3ex9R | GCCCAGCCAGGTTTATTATCTATC |
| TNPO3ex10F | AAATACCCTCGTGATTTAGGCAAC |
| TNPO3ex10R | GATGGAACATTCACAGAAGACACAG |
| TNPO3ex11F | TGTTTTGTTTGTTTTGGCATTCTG |
| TNPO3ex11R | AAAATAGTGTCATTTTCCTTCCTCTTG |
| TNPO3ex12F | TTTGGCCAGAATATAACCTCTGAAATC |
| TNPO3ex12R | ACAGAAAGGAAAACACATGCAAAAG |
| TNPO3ex13F | GGGCCTGTTGACAATATGTAAGTG |
| TNPO3ex13R | TGACAGATGTAAGATTACCCCAGGT |
| TNPO3ex14F | AAAAGAATGGAACCAAACCAGTTC |
| TNPO3ex14R | GTGCATTTTCTTGGATTTTCTAGG |
| TNPO3ex15F | TGTATGTTGCAGTGTTGGCTGTAG |
| TNPO3ex15R | ATGGTTTCATTTGAGCCTTTAACC |
| TNPO3ex16F | CTAAGTAAGCAAGCAGGGATCAGG |
| TNPO3ex16R | GTGAGTTACCCAAAGCCCTGTAAG |
| TNPO3ex17F | GCTGGCCATGTGTGTTCCTATAC |
| TNPO3ex17R | GCACCCTGCCTATTTCAAATTC |
| TNPO3ex18F | TCTGAATTTCTTCCTGGAGACAGC |
| TNPO3ex18R | ATGGTAAAGAATACGCTGCTAGGC |
| TNPO3ex19F | GCTGTGCTTTTCTTCTTTGTTCCT |
| TNPO3ex19R | CTTGCAATTTACCTTTGGGTTATG |
| TNPO3ex20F | CTTCATAGTTTTGCCCTTTTACCC |
| TNPO3ex20R | ATGCTGGTTTTCTCCTAAGGAATG |
| TNPO3ex21F | TACTAAAGGGGAAAGAGTGCTTGG |
| TNPO3ex21R | GGCTAGTTTCAAAATTAGGGTTGG |
| TNPO3ex22F | CTAAGGAAAATTTGTGGGGTTTTG |
| TNPO3ex22R | CCCAAGGGCACATAAGAAATAAAG |
| TNPO3ex23F | GATGGAGAGAGCTCCTAGCAGAG |
| TNPO3ex23R | AGGCATTAAAACCTATCCCCATTC |
| TNPO3ex24F | TTATTGGCAGACTTCAGAATGAGG |
| TNPO3ex24R | TTTCACAGAAAGCTTGTCCTTTTG |

| **cDNA primers** | |
| --- | --- |
| TNPO3cDNA1F | GACAGTGAGGAAGCGCGAAG |
| TNPO3cDNA1R | AGCAAAATAGCATGACTCCACATC |
| TNPO3cDNA2F | CTTAGTCCAAGCCTGGATTTCAGT |
| TNPO3cDNA2R | ATTAGCTCCAATTCGTAAGGAACG |
| TNPO3cDNA3F | CAATAGCAGATCTTGCCCTACAG |
| TNPO3cDNA3R | AAGCAGCTTCATGTAGGTTAGACG |
| TNPO3cDNA4F | GTTGGTTTAACTTGGGAGTTTTGG |
| TNPO3cDNA4R | AAACTCCCCAAAGTCATCAGTCTC |
| TNPO3cDNA5F | GGCATCTTCAAAGCTTACATTCAG |
| TNPO3cDNA5R | AGCAAATGTTATGAATGGCTTTGG |
| TNPO3cDNA6F | GATCGAAATCCTCAGTTCCTTGAC |
| TNPO3cDNA6R | ACCTGCAACAACGCTCTACAATC |
| TNPO3cDNA7F | GACTCATCCGTGTCAGAAAGTCATAC |
| TNPO3cDNA7R | GGCTACCCCTGTATGAATGAGGTC |
| TNPO3cDNA8F | GCCAAGTGGTCATCCCTATCTTAC |
| TNPO3cDNA8R | GATTGTGGGACACAGTCTGGTT |
