# Supplementary material for: Next-Generation Sequencing Identifies Transportin 3 as the Causative Gene for LGMD1F
Source: PLoS One. 2013 May 7;8(5):e63536. doi: 10.1371/journal.pone.0063536 (PMC3646821; doi:10.1371/journal.pone.0063536)
Supplement: Table S3 — Exons inadequately covered by NGS exome sequencing and primers designed for Sanger sequencing. (DOC) [file pone.0063536.s003.doc]

**Table S3:** Exons inadequately covered by NGS exome sequencing and primers designed for Sanger sequencing

| **Primers sequences** | | **Human genomic coordinates (hg.19)** |
| --- | --- | --- |
| PRRT4_I F | GGAAGGAATCTGGAGAGGTATCTG | chr7:127,990,939-127,993,041 |
| PRRT4_I R | CAGCGATTACACCGTGGACTTC |
| PRRT4_II F | CCGCCCTGGAGTAGGAGGTCTGG |
| PRRT4_II R | CCTATGGGCACAGGGATCGACTG |
| PRRT4_III F | GCCCATAGGCGTCGTAGAAGAG |
| PRRT4_III R | CCTATGGGCACAGGGATCGACTG |
| PRRT4_IV F | GGCACGTAGAGTGTTCCACAATAG | chr7:127,999,095-128,000,146 |
| PRRT4_IV R | AGTGAGGCCTCTATGCTGTCTCTC |
| PRRT4_V F | CTCAGTGCCATGTCAAACTTCAG |
| PRRT4_V R | CTGAGATCAGAAGCCTCTCTCTGG |
| PRRT4_VI F | ACACACACACACGGCATGTACTG | chr7:128,001,305-128,001,943 |
| PRRT4_VI R | CTGGGAGTGTGTGATTGCTCTCGC |
| IMPDH1_I F | GGCCTGGCAGAGAGAGTACTTG | chr7:128,036,654-128,036,774 |
| IMPDH1_I R | ATGATGGGCTTTGGTCACTGTA |
| IMPDH1_II F | GTACGAGACCCGGCGCTTACTTG | chr7:128,045,643-128,046,167 |
| IMPDH1_II R | GTCCTGGGTGGCTGGATGTAGAG |
| IMPDH1_III F | CTCTCAGATCTCAGTGCATGGTG | chr7:128,049,358-128,049,710 |
| IMPDH1_III R | CCTATTACCGCCTCAGCCTCTAAC |
| CALU_I F | CAAGTATTTCGCTGGTTCCTAATC | chr7:128,388,353-128,388,482 |
| CALU_I R | GCATCATTGTGAACCTTGTCACTG |
| CCDC136_I F | AAACAGTCCCACCTCGTCCTTTG | chr7:128,431,208-128,431,732 |
| CCDC136_I R | GGATGGAGGGAGGAAGACTTTGG |
| FLNC_I F | GTGTAAAGCCACAGCCTCTGAG | chr7:128,477,275-128,477,312 |
| FLNC_I R | GGTCATAACAGAATGCTCATCCAC |
| FLNC_II F | CAGGAGGATGAACACCCAAATTATC | chr7:128,489,953-128,490,178 |
| FLNC_II R | CTGCAAGAGAAGGAGTGCTGTGC |
| FLNC_III F | GCTAGGAGGAATCCCAGTGTTGCC | chr7:128,494,516-128,494,763 |
| FLNC_III R | CAAGGTAGTGGACTTCAGCATCATC |
| LOC130705_I F | AAGTAGAACCCTGGCCCTGTG | chr7:128506463-128506884 |
| LOC130705_I R | CCTAGTTCTGTGGAACGTGTCG |
| LOC130705_II F | CCATTCCTCCAAAGCACTTTCTAT | chr7:128508720-128508829 |
| LOC130705_II R | GATTAAAGTGATGAGGGTTACGG |
| KCP_I F | CCAGCACCAGGAGGAAATACAC | chr7:128,516,811-128,517,511 |
| KCP_I R | CAGTTTCAGGTGCCTTGGTAAC |
| KCP_II F | CTCCCAGAAAACCAAGCATCTC | chr7:128,520,374-128,520,635 |
| KCP_II R | CAGAACAATGATGTCTGTCCTCAG |
| KCP_III F | GCAGCCCTAACCTCATATCTCATC | chr7:128,524,040-128,524,200 |
| KCP_III R | GGAGCACGTCTAGAAGACACTGAG |
| KCP_IV F | CACAGGGATACACTCCTTTGGCC | chr7:128,524,614-128,525,324 |
| KCP_IV R | CCCTCCACAACAGACAGGAGTTC |
| KCP_V F | CTATACCTGACAGCGACACCACTC | chr7:128,526,844-128,527,105 |
| KCP_V R | GCTACACTCTGCCCTTCACATCTC |
| KCP_VI F | CTCTATCAAAGGTGAGGGAGTCG | chr7:128,528,883-128,529,131 |
| KCP_VI R | CCAAGCTCAGAGCCTATGACCAC |
| KCP_VII F | GCTGACCGGACACTACATTCCTAAC | chr7:128,530,866-128,531,548 |
| KCP_VII _R | CTCCCCCAACTAACCCACTGACCCG |
| KCP_VII bis_F | ACCACGGGTCAGTGGGTTAGTTG |
| KCP_VII bis_R | ATCCTGGAGGAAGAGGTGTTTGTG |
| KCP_VIII F | GACTCACTTTGGGAACAGGGTAAC | chr7:128,533,712-128,534,300 |
| KCP_VIII R | GGATTCTAATGCCATTTCCACCTC |
| KCP_IX F | GAACCTCAGGCAGGGCTAAGAG | chr7:128550654-128550730 |
| KCP_IX R | TGGGTTTATTTTCTTTTCAGGGTC |
| TSPAN33_I F | AGAGGGATGGCTGGATACTTAGTG | chr7:128,784,609-128,784,935 |
| TSPAN33_I R | CAGGATAAATTCCCCCAAAGTG |
