# Supplementary material for: Next-Generation Sequencing Identifies Transportin 3 as the Causative Gene for LGMD1F
Source: PLoS One. 2013 May 7;8(5):e63536. doi: 10.1371/journal.pone.0063536 (PMC3646821; doi:10.1371/journal.pone.0063536)
Supplement: Table S4 — Co-segregation study. (DOC) [file pone.0063536.s004.doc]

**Table S4:** Co-segregation study

| **Phenotype** | **Pedigree position** | ***Alu I* digestion** | **Sanger sequencing** |
| --- | --- | --- | --- |
| Not affected | V-29 | YES complete | WT/WT |
| Not affected | VI-52 | YES complete | WT/WT |
| Not affected | VI-58 | YES complete | WT/WT |
| Not affected | VI-59 | YES complete | WT/WT |
| Not affected | VI-61 | YES complete | WT/WT |
| Not affected | VI-63 | YES complete | WT/WT |
| Not affected | VII-22 | YES complete | WT/WT |
| Not affected | VII-23 | YES complete | WT/WT |
|  |  |  |  |
| Affected | V-8 | 50% | WT/delA p.X924C |
| Affected | V-12 | 50% | WT/delA p.X924C |
| Affected | V-14 | 50% | WT/delA p.X924C |
| Affected | V-18 | 50% | WT/delA p.X924C |
| Affected | V-19 | 50% | WT/delA p.X924C |
| Affected | V-25 | 50% | WT/delA p.X924C |
| Affected | V-28* | 50% | WT/delA p.X924C |
| Affected | VI-11 | 50% | WT/delA p.X924C |
| Affected | VI-27 | 50% | WT/delA p.X924C |
| Affected | VI-36** | 50% | WT/delA p.X924C |
| Affected | VI-53* | 50% | WT/delA p.X924C |
| Affected | VI-56 | 50% | WT/delA p.X924C |
| Affected | VI-57 | 50% | WT/delA p.X924C |
| Affected | VI-60 | 50% | WT/delA p.X924C |
| Affected | VII-5* | 50% | WT/delA p.X924C |
| *Sequenced by Solid  ** Sequenced by Illumina | |  | |
