# Supplementary material for: Next-Generation Sequencing Identifies Transportin 3 as the Causative Gene for LGMD1F
Source: PLoS One. 2013 May 7;8(5):e63536. doi: 10.1371/journal.pone.0063536 (PMC3646821; doi:10.1371/journal.pone.0063536)
Supplement: Table S5 — Alignment of Human and Danio r. TNPO3 proteins. (DOC) [file pone.0063536.s005.doc]

**Table S5:** Alignment of Human and *Danio r.* TNPO3 proteins

Human 1 MEGAKPTLQLVYQAVQALYHDPDPSGKERASFWLGELQRSVHAWEISDQLLQIRQDVESC 60

MEG KPTL LVYQAVQALYHDPDP+GKERAS WLGELQRS++AWEISDQLLQ++QD+ESC

Danio 1 MEGGKPTLPLVYQAVQALYHDPDPAGKERASVWLGELQRSMYAWEISDQLLQLKQDIESC 60

Human 61 YFAAQTMKMKIQTSFYELPTDSHASLRDSLLTHIQNLKDLSPVIVTQLALAIADLALQMP 120

YFAAQTMKMKIQTSFYELP DSH +LRDSLL+HIQNLKDLSP+IVTQLALAIADLALQM

Danio 61 YFAAQTMKMKIQTSFYELPPDSHTALRDSLLSHIQNLKDLSPIIVTQLALAIADLALQMA 120

Human 121 SWKGCVQTLVEKYSNDVTSLPFLLEILTVLPEEVHSRSLRIGANRRTEIIEDLAFYSSTV 180

SWKGCV TL+EKYSNDV+S+ FL+EILTVLPEEVHSRSLRIGANRRTEIIEDLA+YS+TV

Danio 121 SWKGCVHTLIEKYSNDVSSMTFLIEILTVLPEEVHSRSLRIGANRRTEIIEDLAYYSTTV 180

Human 181 VSLLMTCVEKAGTDEKMLMKVFRCLGSWFNLGVLDSNFMANNKLLALLFEVLQQDKTSSN 240

V+LL+TC EK+G DEKML+KVFRCLGSWFNLGVLD+NFMANN+LL +LF+VLQ+D+TS+N

Danio 181 VTLLVTCAEKSGHDEKMLIKVFRCLGSWFNLGVLDNNFMANNQLLMILFQVLQRDETSTN 240

Human 241 LHEAASDCVCSALYAIENVETNLPLAMQLFQGVLTLETAYHMAVAREDLDKVLNYCRIFT 300

LHEAASDCVCSALYAIENV +LPLAMQLFQGVLTLETAYHMAVAREDLDKVLNYCRIFT

Danio 241 LHEAASDCVCSALYAIENVAIHLPLAMQLFQGVLTLETAYHMAVAREDLDKVLNYCRIFT 300

Human 301 ELCETFLEKIVCTPGQGLGDLRTLELLLICAGHPQYEVVEISFNFWYRLGEHLYKTNDEV 360

ELCETFLE V TPGQG+GDLRTLELLLICAGHPQYEVVEISFNFWYRLGEHLYK ND

Danio 301 ELCETFLEMTVRTPGQGMGDLRTLELLLICAGHPQYEVVEISFNFWYRLGEHLYKINDPA 360

Human 361 IHGIFKAYIQRLLHALARHCQLEPDHEGVPEETDDFGEFRMRVSDLVKDLIFLIGSMECF 420

+H +F+ YIQRLLH+LARHCQL+PDHEGVPE+TDDFGEFRMRVSDLVKD+IFL+GSMECF

Danio 361 LHNVFRPYIQRLLHSLARHCQLDPDHEGVPEDTDDFGEFRMRVSDLVKDVIFLVGSMECF 420

Human 421 AQLYSTLKEGNPPWEVTEAVLFIMAAIAKSVDPENNPTLVEVLEGVVRLPETVHTAVRYT 480

AQLYSTL+EGNPPWEVTEAVLFIMA+IAK+VDPENNPTL+EVLE +V LPETVH AVRYT

Danio 421 AQLYSTLREGNPPWEVTEAVLFIMASIAKNVDPENNPTLMEVLEQIVLLPETVHLAVRYT 480

Human 481 SIELVGEMSEVVDRNPQFLDPVLGYLMKGLCEKPLASAAAKAIHNICSVCRDHMAQHFNG 540

SIELVGEMSEV+DRNP LD VL +LMKGL EKPLASAAAKAIHNICSVCRDHMAQHF G

Danio 481 SIELVGEMSEVIDRNPSMLDTVLNFLMKGLREKPLASAAAKAIHNICSVCRDHMAQHFQG 540

Human 541 LLEIARSLDSFLLSPEAAVGLLKGTALVLARLPLDKITECLSELCSVQVMALKKLLSQEP 600

LL+IARSLDSF LS +AAVGLLKGTALVLARLPL+KI ECL++LC+VQVMALKKLL+Q+

Danio 541 LLDIARSLDSFALSTDAAVGLLKGTALVLARLPLEKIAECLNDLCAVQVMALKKLLAQDS 600

Human 601 SNGISSDPTVFLDRLAVIFRHTNPIVENGQTHPCQKVIQEIWPVLSETLNKHRADNRIVE 660

S+G SSDPTV+LDRLAVIFRHTNPIVENGQTHPCQKVIQEIWPVLSETLN H++DNRIVE

Danio 601 SSGKSSDPTVWLDRLAVIFRHTNPIVENGQTHPCQKVIQEIWPVLSETLNAHQSDNRIVE 660

Human 661 RCCRCLRFAVRCVGKGSAALLQPLVTQMVNVYHVHQHSCFLYLGSILVDEYGMEEGCRQG 720

RCCRCLRFAVRCVGKGSA+LLQPLVTQMV+VY ++ HSCFLYLGSILVDEYGMEEGCRQG

Danio 661 RCCRCLRFAVRCVGKGSASLLQPLVTQMVSVYQLYPHSCFLYLGSILVDEYGMEEGCRQG 720

Human 721 LLDMLQALCIPTFQLLEQQNGLQNHPDTVDDLFRLATRFIQRSPVTLLRSQVVIPILQWA 780

LLDMLQALC+PTFQLLEQ NGL+NHPDTVDDLFRLATRF+QRSPVTLL S +++ I+Q A

Danio 721 LLDMLQALCMPTFQLLEQPNGLRNHPDTVDDLFRLATRFVQRSPVTLLSSSIIVHIIQCA 780

Human 781 IASTTLDHRDANCSVMRFLRDLIHTGVANDHEEDFELRKELIGQVMNQLGQQLVSQLLHT 840

IA+TTLDHRDANCSVM+F+RDLIHTGV NDHE+DFE+RK LIGQ M Q GQQLV+QL++T

Danio 781 IAATTLDHRDANCSVMKFIRDLIHTGVTNDHEDDFEVRKRLIGQAMEQHGQQLVNQLINT 840

Human 841 CCFCLPPYTLPDVAEVLWEIMQVDRPTFCRWLENSLKGLPKETTVGAVTVTHKQLTDFHK 900

CCFCLPPYTLPDVAEVLWEIM DRPTFCRWLE +LKGLPKET GAVTVTHKQLTDFHK

Danio 841 CCFCLPPYTLPDVAEVLWEIMVFDRPTFCRWLETTLKGLPKETAGGAVTVTHKQLTDFHK 900

Human 901 QVTSAEECKQVCWALRDFTRLFR 923

QVTSAEECKQVCWA+R+FTRL+R

Danio 901 QVTSAEECKQVCWAIREFTRLYR 923
